# Jejunal Ectopic Pancreas: A Rare Cause of Small Intestinal Mass

**DOI:** 10.7759/cureus.15409

**Published:** 2021-06-02

**Authors:** Samyak Dhruv, Abhishek Polavarapu, Ifeyinwa Asuzu, Sherif Andrawes, Indraneil Mukherjee

**Affiliations:** 1 Internal Medicine, Northwell Health, New York, USA; 2 Gastroenterology, Northwell Health, New York, USA; 3 Pathology, Northwell Health, New York, USA; 4 Surgery, Northwell Health, New York, USA

**Keywords:** ectopic, pancreas, small intestine, mass, jejunum

## Abstract

Ectopic pancreas (EP) is defined as the presence of pancreatic tissue outside the pancreas with no anatomical connection to the pancreas. It is also known as pancreatic heterotopia, accessory pancreas, aberrant pancreas, or pancreatic rest. The first case of EP was described in 1727 when pancreatic tissue was identified in the ileal diverticulum. Abdominal pain and lower gastrointestinal bleeding are the most common symptoms. On histopathological examination, EP can be classified into four subtypes. Once identified and diagnosed, the treatment is surgical resection and the post-operative course is asymptomatic in most cases. We describe a rare case of EP, which was discovered on the CT scan of the abdomen as a jejunal mass and successfully treated with surgical resection. We have also described the possible role of chromogranin A for diagnosing EP cases and for post-operative follow-up.

## Introduction

The first case of ectopic pancreas (EP) or pancreatic heterotopia was described in 1727 when it was identified in the ileal diverticulum [1]. It is a rare congenital anomaly, in which there is a development of the pancreatic tissue outside the pancreas without any ductal or vascular connection to the main pancreatic tissue [2]. It is most commonly found in the stomach (particularly antrum), duodenum, jejunum, or in a Meckel diverticulum. Other locations where EP has been identified are the ileum, liver, spleen, biliary tract, mesentery, fallopian tubes, or umbilicus [3]. It is mostly located in the submucosa but rarely can be found in the muscularis or serosa. The incidence of EP on the autopsy specimens is around 0.5%-13.7% with male predominance [4]. It is often asymptomatic and in most cases found incidentally during the surgery done for the other indications [3]. We present a rare case of the jejunal mass identified to be an EP.

## Case presentation

**Figure 1:**
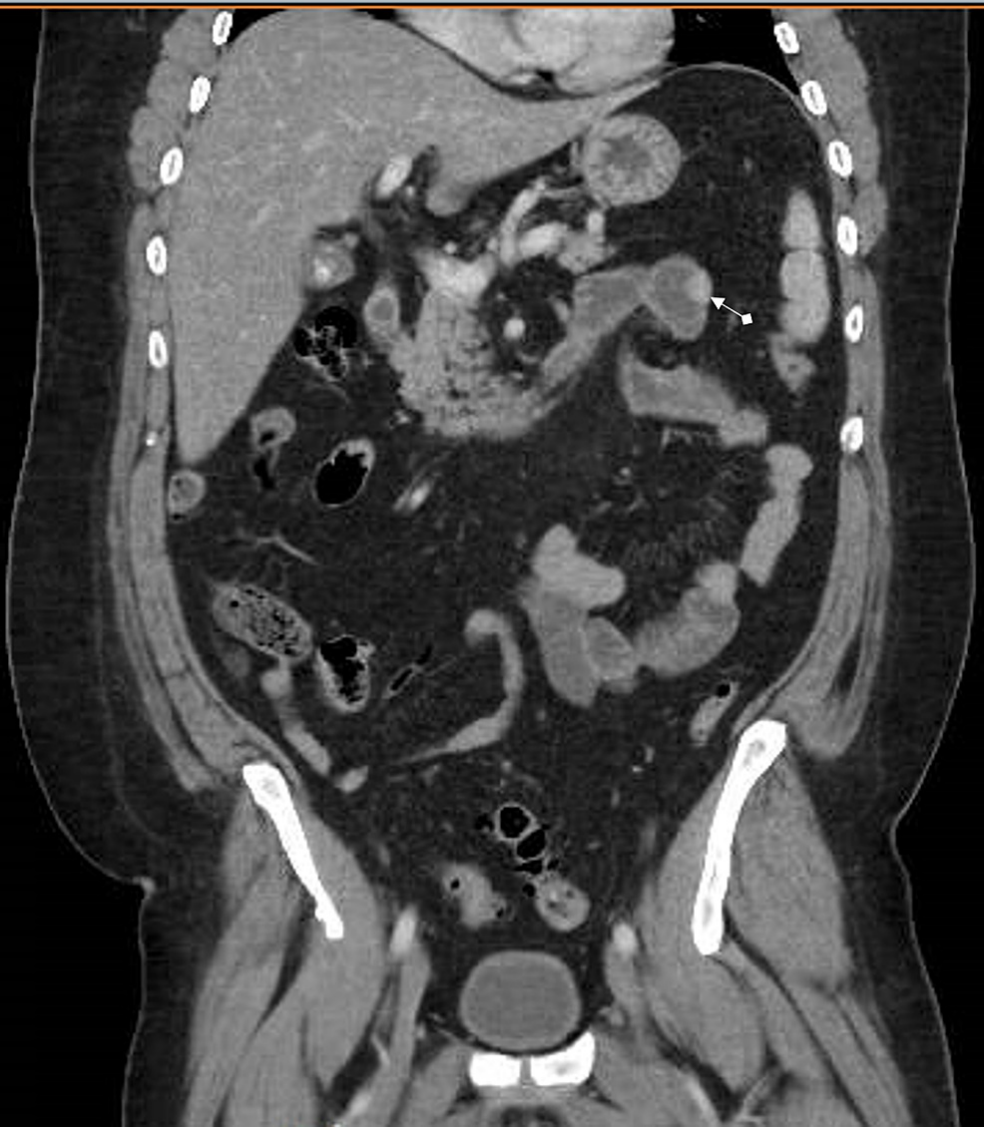
Coronal section of CT abdomen with IV contrast showing 1.5 cm hyperdense, circular jejunal lesion with regular borders in the left upper quadrant.

**Figure 2:**
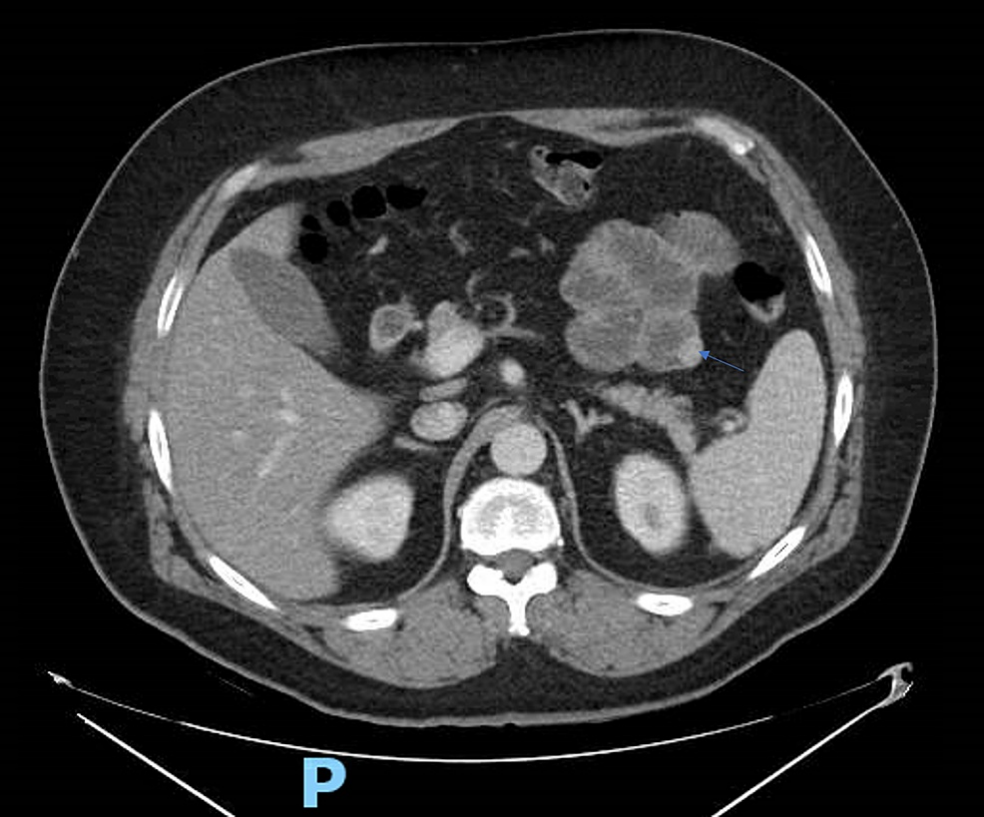
Axial section of CT abdomen with IV contrast showing 1.5 cm hyperdense, circular jejunal lesion with regular borders.

**Figure 3:**
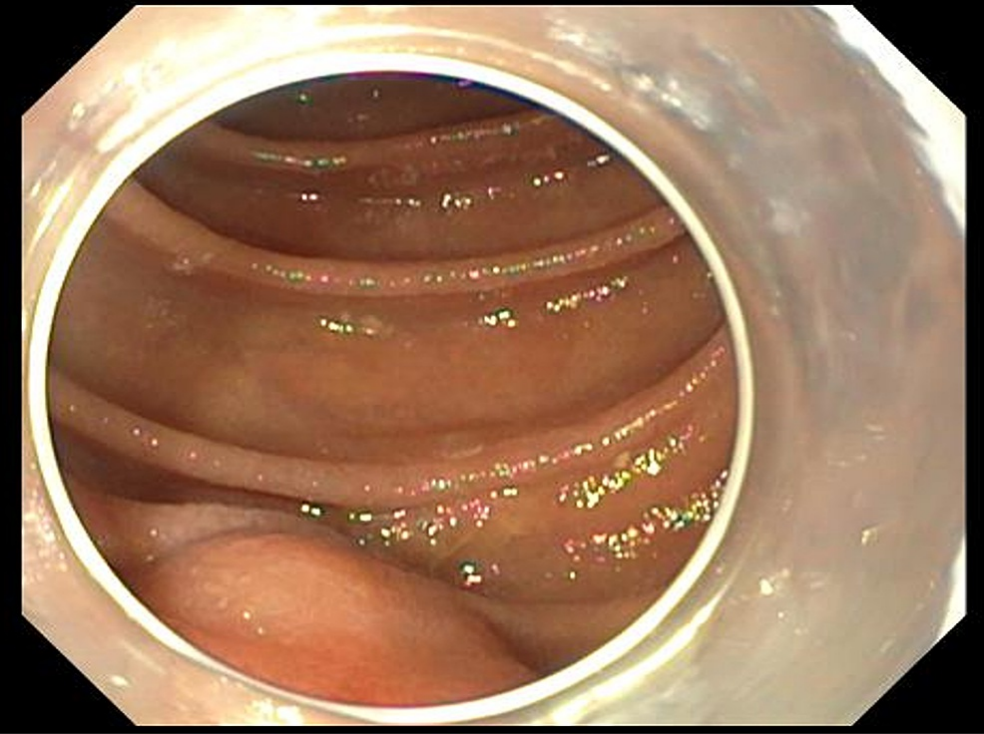
Single balloon enteroscopy with distal cap attachment showing a 1.5 cm smooth regular submucosal lesion in the proximal jejunum.

**Figure 4:**
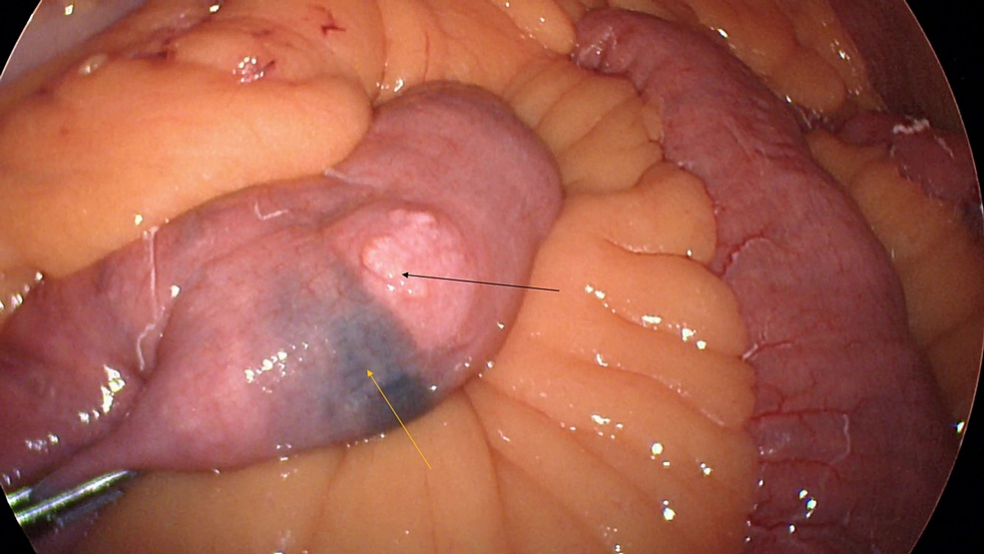
Laparoscopic image of jejunum showing the mass (black arrow) and site of the ink injection done during previous endoscopy (yellow arrow).

**Figure 5:**
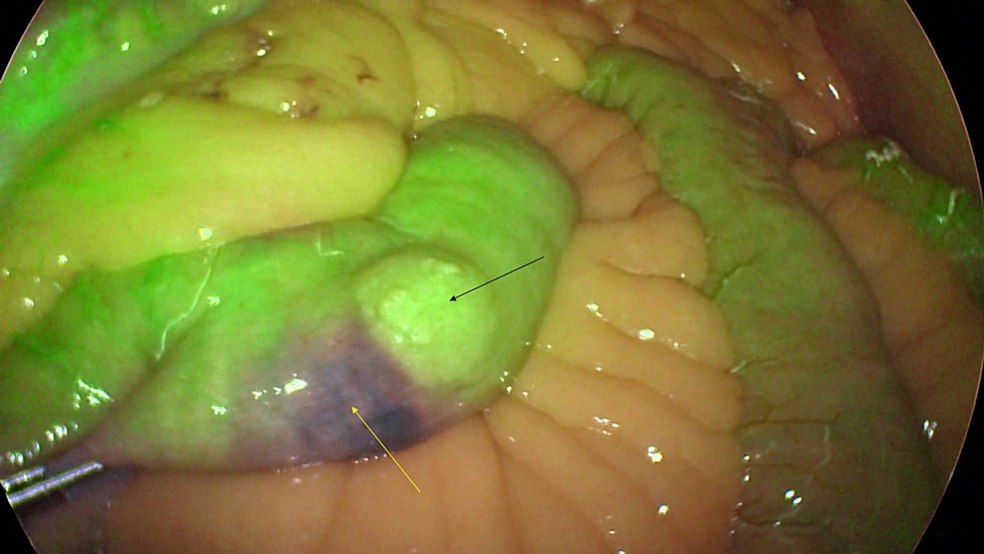
ICG dye overlay mode with near-infrared fluorescence demonstrating better demarcation of the lesion (black arrow) and the site of ink injection done during previous endoscopy (yellow arrow). ICG, indocyanine green

**Figure 6:**
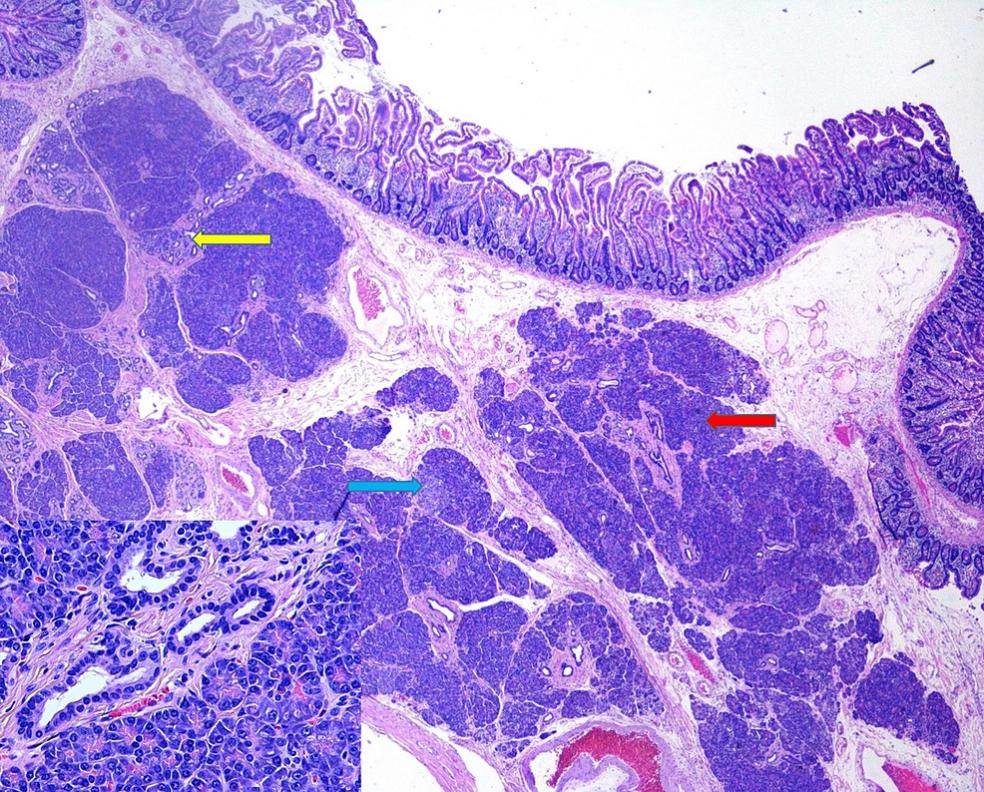
Small intestinal wall with lobules of benign pancreatic acini (red arrow), ducts (yellow arrow), and rare islets (blue arrow) within the submucosa, consistent with type 1 EP according to Heinrich classification (pancreatic heterotopia). H&E, 20X. EP, ectopic pancreas

A 48-year-old male with a past medical history of hypertension and diabetes presented to the emergency room (ER) with left lower quadrant stabbing pain which was intermittent, moderate to severe with no worsening or relieving factors. He denied any fever, chest pain, shortness of breath, nausea, vomiting, dysuria, hematuria, diarrhea, or constipation. His last bowel movement was on the day of the admission. The patient was a nonalcoholic and a nonsmoker. There was no family history of any pancreatic or gastrointestinal cancers. The pain was present for one year before the admission and had worsened two days before the ER visit. Vital signs on admissions were: temperature 97.9°F, heart rate 84 beats/min, blood pressure 170/92 mmHg, respiratory rate 14/min, and oxygen saturation 99% on room air. On physical examination, the abdomen was soft, nontender, and nondistended without any guarding or rigidity. There was no evidence of swelling or mass. Digital rectal examination was negative for any signs of blood. Laboratory studies including lipase, amylase, and lactate were normal. CT scan of the abdomen with IV contrast showed a 1.5 cm mass in the proximal loop of the jejunum with contrast enhancement (Figures 1-2). His abdominal pain was thought to be due to the mass. The pain resolved in few hours and he was discharged with an outpatient follow-up with the gastroenterology team for a single balloon enteroscopy to further investigate the mass. He underwent a single balloon enteroscopy with the gastroenterology team a few days later. The scope was advanced to the distal jejunum and then pulled back while carefully examining the folds of the jejunum. A 1-2 cm subepithelial lesion was identified in the proximal jejunum with a small umbilication on top of the lesion (Figure 3). He underwent a biopsy of the mass. Biopsy results showed preserved small bowel villous architecture without any evidence of inflammation or malignancy. His abdominal pain reappeared and resection of the unexplained mass was considered by the surgery team. A repeat CT scan of the abdomen was performed, which showed the jejunal mass in the same location without a change in size. His carcinoembryonic antigen (CEA) and cancer antigen 19-9 (CA 19-9) levels were normal. Chromogranin A levels were elevated to 318.1 ng/mL. He was, therefore, scheduled for the laparoscopic resection of the mass. He underwent laparoscopic resection of the small bowel mass (Figures 4-5). We used indocyanine green (ICG) dye as the mass looked hypervascular on the CT scan images. Though, in ICG dye images we did not see any difference between the mass and the surrounding structures suggesting that the cells of the lesion had a similar blood supply and metabolism as the surrounding tissues. It did help to show a more circular lesion (Figure 5) compared to the image without the dye where the margins of the mass look very irregular (Figure 4). A small segment of the small bowel which had the mass was removed by segmental resection and side-to-side anastomosis was performed with a stapler. Histopathological analysis showed the mass as the ectopic pancreatic tissue (Figure 6). He was started on a liquid diet the next morning and was discharged a day later. At his last follow-up one month after the surgery, he remained symptom-free. His chromogranin A levels declined to 156.8 ng/mL. Currently, he is being followed by a general surgery team with repeat measurements of chromogranin A levels every month to ensure normalization of the chromogranin A levels.

## Discussion

The true incidence of EP is not known as most patients are asymptomatic. In the majority of cases, EP is identified incidentally during the surgery performed for other indications [5]. When symptomatic due to local inflammation, patients may develop abdominal pain, pancreatitis, gastrointestinal bleeding, intestinal obstruction, or perforation. Rare cases of premalignant EP tissues have also been reported [6]. Due to the lack of established surveillance guidelines, these lesions cause a dilemma in surveillance even after complete resection. The development of malignancies such as adenocarcinoma, intraductal papillary mucinous neoplasm, and the solid pseudopapillary tumor is extremely rare, although reported in the literature [7]. There are various theories behind the development of EP. According to one theory, duodenal evagination persists after the development of normal pancreatic tissue [8]. This evagination then separates from the actual pancreatic tissue and migrates along the developing gastrointestinal tract, which could explain the different locations of EP. Another theory suggests pancreatic metaplasia of the endodermal tissues in the gastric mucosa, which could explain the location of EP in the stomach [8].

Pre-operative diagnosis of EP is extremely difficult though high clinical suspicion can help. It appears as an endoluminal enhancing lesion on the CT scan. On endoscopy, it appears as a round to oval submucosal lesion with central ulceration or umbilication [9]. Definitive diagnosis is only made by histopathological examination. It can show pancreatic ducts, acini, and/or islet cells on histology. EP was classified into three subtypes according to their histopathological findings by Heinrich in 1909 [10]. Type 1 (most common) contains acini, pancreatic ducts, and islet cells; type 2 is composed of acini and pancreatic ducts without islet cells; type 3 has only pancreatic ducts. Type 4 was later added by Gasper in 1973 which contains only pancreatic islet cells on histological examination [10]. Our case is an example of a type 1 EP. Endoscopic diagnosis is rarely conclusive on biopsies as the lesion in most cases is submucosal and biopsy tissues are rarely deep enough to reach the tumor [11]. In our case also biopsies done during enteroscopy were not conclusive. Surgery is indicated in symptomatic cases as well as in incidentally discovered cases to prevent further future complications related to EP tissue [12]. Chromogranin A level was measured pre-operatively in our case to further categorize the mass, which was elevated and declined post-operatively. There are no case reports in the literature that mentions any diagnostic value of chromogranin A in EP. Our case suggests that chromogranin A levels can be used to diagnose EP and can be followed up post-operatively to confirm the complete resection of the potentially malignant EP tissue. Though the level of chromogranin A can be nonspecific, it can be seen elevated in several pathologies including neuroendocrine carcinoma, atrophic gastritis, and chronic use of acid-suppressive medications [13]. Recurrence is not noted and generally, patients remain asymptomatic after the surgical removal.

## Conclusions

Ectopic pancreas should be considered in the differential diagnosis of small bowel tumors whether identified on the imaging studies or incidentally discovered during surgery performed for other reasons. Pre-operative presumptive diagnosis requires a high degree of clinical suspicion. Survival after resection is good and the post-operative course is generally asymptomatic, highlighting the importance of the early diagnosis of this rare entity. It is important that radiologists, gastroenterologists, surgeons, and oncologists are aware of this rare entity to prevent the delay in care and appropriate management.
